# Evaluation of the safety of tranexamic acid use in pediatric patients undergoing spinal fusion surgery: a retrospective comparative cohort study

**DOI:** 10.1186/s12891-022-05604-2

**Published:** 2022-07-08

**Authors:** Iryna Ivasyk, Abhinaba Chatterjee, Catherine Jordan, Matthew T. Geiselmann, Peter S. Chang, Hooman Kamel, Sariah Khormaee

**Affiliations:** 1 Weill Cornell Medical College, New York, NY USA; 2 Department of Psychology, Thompson Rivers University, Kamloops, BC Canada; 3 New York Institute of Technology College of Osteopathic Medicine, Old Westbury, New York, USA; 4 Department of Orthopedic Surgery, Washington University, St. Louis, MO USA; 5 Clinical and Translational Neuroscience Unit, Feil Family Brain and Mind Research Institute and Department of Neurology, Weill Cornell Medicine, New York, NY USA; 6 Hospital for Special Surgery, New York, NY USA

**Keywords:** Tranexamic acid, Txa, Spinal fusion, Antifibrinolytic, Pediatric

## Abstract

**Background:**

Pediatric spinal fusion may be associated with significant intraoperative blood loss, leading to complications from transfusion, hypoperfusion and coagulopathy. One emerging strategy to mediate these risks is by utilization of the anti-fibrinolytic agent tranexamic acid (TXA). However, concerns regarding potential adverse reactions, specifically postoperative seizures and thrombotic events, still exist. To assess these risks, we examined the perioperative morbidity of TXA use in a large national database.

**Methods:**

Retrospective data from pediatric patients (age 18 years or younger), discharged between January 2013 to December 2015, who underwent primary or revision posterior spinal fusions, was collected from the Premier Perspective database (Premier, Charlotte, NC). Patients were stratified by TXA use and records were assessed for complications of new onset seizures, strokes, pulmonary embolisms (PE) or deep vein thromboses (DVT) occurring during the perioperative period.

**Results:**

In this cohort of 2,633 pediatric patients undergoing posterior spinal fusions, most often to treat adolescent idiopathic scoliosis, 15% received TXA. Overall, adverse events were rare in this patient population. The incidence of seizure, stoke, PE, or DVT in the control group was 0.54% (95% CI, 0.31% to 0.94%) and not significantly different from the TXA group. There was no significant difference in the incidence of DVTs, and no incidences of stroke in either group. There were no new-onset seizures or PEs in patients who received TXA.

**Conclusions:**

The use of TXA was not associated with an increased risk of adverse events including seizure, stroke, PE, and DVT. Our findings support the safety of TXA use in pediatric patients undergoing spinal fusion surgery.

## Background

Significant intraoperative blood loss occurring during pediatric spinal fusion may lead to complications associated with transfusion, hypoperfusion, and coagulopathy [1, 2]. One way to effectively decrease intraoperative blood loss is through the use of tranexamic acid (TXA), an antifibrinolytic agent [3–5]. This medication has been extensively used in cardiac surgery [6–8] and other types of orthopedic surgery [9–13], including spine surgery [14, 15]. Two studies, with large sample sizes of 1,769 and 4,269 pediatric patients undergoing spinal surgery assessed TXA use, but their findings were limited to TXA efficiency in decreasing blood loss without an analysis of adverse events associated with the drug [16, 17]. A few smaller studies, with sample sizes ranging from 44 to 166 patients, reported on the safety of TXA use in this population and did not find any associated adverse events [18–25]. However, concerns remain that rare, yet serious side effects, may not be adequately captured in a small sample.

**Fig. 1:**
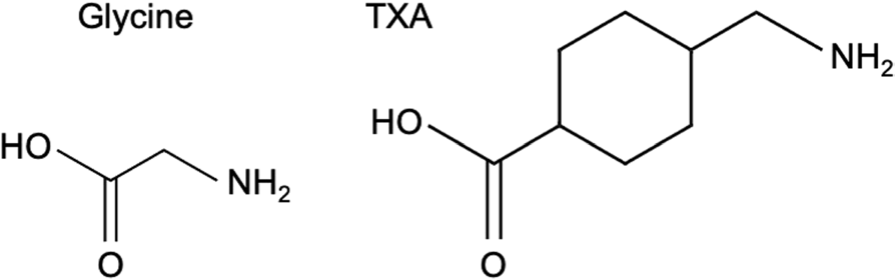
Chemical structures of glycine, an excitatory neurotransmitter and TXA

Due to similarity in molecular structure, TXA may act as a competitive inhibitor of glycine (Fig. 1), and studies in animal models have demonstrated that competitive inhibition of glycine receptors by TXA could lead to excitation of neural tissues and result in seizure [26–28]. Some studies have found a link between TXA use and seizures in non-primate model organisms [29], and a few case reports have demonstrated this epileptic potential in humans [30–32]. Perhaps most importantly, multiple studies in adult patients found an increased risk of seizure after TXA use in cardiac surgery [33, 34]. However, the risk of this adverse event after TXA use in pediatric spine surgery is still unclear [6, 35, 36]. Additionally, due to its function as an anti-fibrinolytic agent, one concern of TXA use is the possibility of increased major thrombotic events such as pulmonary embolism (PE), deep vein thrombosis (DVT), and stroke. This is more frequently explored in the literature.

To address these concerns of TXA safety in pediatric spine fusion patients, we used a large national database to evaluate the association between TXA use and new-onset postoperative thrombotic events or seizures.

## Methods

### Data source

This study was a retrospective cohort analysis of the Premier Perspective Database (Premier, Charlotte, NC), which sources data from approximately 600 hospitals across the United States. This database is anonymized and contains encrypted identifiers to link longitudinal data from a single patient. Other data available include patient demographics, diagnostic/procedural coding information, including distinct codes for new onset diagnoses and those prior to admission, and drug administration during inpatient stays. The institutional review board at Weill Cornell Medicine approved this study and exempted the need for informed consent.

### Patient cohort

Our study included patients 18 years or younger who were undergoing primary or revision posterior spinal fusions (ICD-9-CM procedure codes 81.0X or 81.3X) with discharge dates between January 1, 2013 to December 31, 2015. We identified all patients who had received TXA by searching for the term “tranexamic” in the list of the administered pharmaceuticals linked to their admission. Patients receiving any dose of TXA were categorized into the TXA group, and those without a note of the term “tranexamic” in their medication administration record were categorized into the non-TXA group. Charlson Comorbidity Index (CCI) was calculated for patients based on ICD-9-CM diagnosis codes using a previously reported algorithm [37].

### Outcomes

We tracked the major complications of deep vein thromboses (451.X), pulmonary embolisms (415.1X), and seizures (780.39, 345.X), all of which were identified as new onset code identifiers associated with previously published codes for these diagnoses. We also examined a less commonly discussed complication of stroke (433.X1, 436). Additionally, for any patients with a documented history of seizure in a prior hospitalization, a diagnosis of seizure during the posterior spinal fusion admission was not considered “new onset”, even if it was identified with a new onset code modifier as such. Finally, we also examined the rate of mortality during the index hospitalization. For large dataset individual privacy protection, the exact rates of adverse events with fewer than 11 incidences were supressed.

### Statistical analysis

Categorical variable differences were measured by Fisher’s Exact test or Chi-Squared as noted. Continuous variable differences were assessed by the 2-tailed Student *t* test or the Mann–Whitney test of nonnormally distributed data. Confidence intervals for fractions were calculated using the Wilson/Brown method. Odds ratios were calculated using the Baptista-Pike method. Statistical analysis was performed using Stata v14.2 (StataCorp, College Station, TX) and GraphPad Prism v9.0.2 (GraphPad Software, San Diego, California).

## Results

**Fig. 2:**
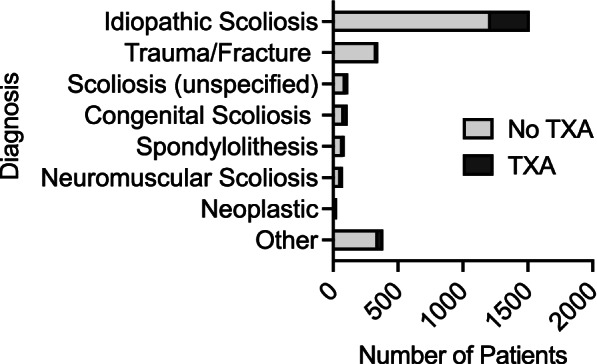
Diagnoses of pediatric spine fusion patients associated with surgical admission

Of the 2,633 pediatric patients who underwent spine fusion in our cohort, 96.2% underwent a primary fusion. The average age in the cohort was 14 ± 3 years; 64.5% were female, and 65.7% were white (Table 1). The most common reason for spinal fusion was a diagnosis of adolescent idiopathic scoliosis (AIS) which was present in 57.5% of patients, followed by traumatic injuries requiring fusion (12.8%). Less frequent (< 5%) primary diagnoses associated with posterior spinal fusion included congenital scoliosis, spondylolisthesis, neuromuscular scoliosis, and neoplastic causes as detailed in Fig. 2.

**Table 1:** Demographics of the pediatric spine fusion population stratified by TXA Use

**Characteristics**	**TXA** (*n* = 402)	**No TXA** (*n* = 2,231)	**All patients** (*n* = 2,633)	***p*-value**
Age (Average ± SD)	14.1 ± 3.11	14.1 ± 2.28	14.1 ± 2.99	0.87
Sex – Female, n (%)	271 (67.4)	1,426 (63.9)	1,697 (64.5)	0.18
Race – White, n (%)	1,449 (65.0)	282 (70.2)	1,731 (65.7)	0.20
Primary Fusion, n (%)	392 (97.5)	2,140 (95.9)	2,532 (96.2)	0.13
CCI (Average ± SD)	0.17 ± 0.38	0.17 ± 0.39	0.17 ± 0.39	0.99

*CCI* Charlson comorbidity index is abbreviated

A fraction of pediatric patients (15.3%, 402 patients) received TXA during their admission for posterior spinal fusion. There was no significant difference in the demographics, including age, sex, and race, or frequency of primary fusion between the two patient populations (Table 1). Patients in both groups had a low Charlton comorbidity index of 0.17.

There were no reported new onset seizures or PE in patients who received TXA perioperatively, and no statistically significant difference in incidence of DVT (Table 2). No strokes were reported in either cohort. There were also no cases of in-hospital mortality.

**Table 2:** Frequency of new onset complications in the pediatric fusion population, stratified by TXA Use.^a^

	**TXA** (*n* = 402)	**No TXA** (*n* = 2,231)	**Odds Ratio**	***P* value**
New onset seizures	0	< 11^b^	0 (0, 3.72) ^c^	> 0.99 ^c^
Pulmonary embolism	0	< 11^b^	0 (0, 5.57)	> 0.99
Deep Vein Thrombosis	< 11 ^b^	< 11 ^b^	1.39 (0.11, 8.35)	0.56
Stroke	0	0	-	-
**Total**	< 11 ^b^	12 (0.31%, 0.94%)	0.46 (0.04, 2.90)	0.71

^a^ 95% confidence intervals shown in parenthesis where applicable. The Fisher exact test was used to calculate p-values

^b^ To protect patient privacy, data in cells with fewer than 11 instances is supressed

^c^ 15 patients with a prior history of seizure were excluded

Overall, new diagnosis of DVT, PE, stroke, and seizure were rare, with no statistically significant difference between the two groups (*p* = 0.71, Table 2). The rate of any complication for patients not receiving TXA was 0.54% (95% CI, 0.31% to 0.94%). The odds ratio of complications in the group that received TXA compared to the group that did not was 0.46 (95% CI, 0.04 to 2.9).

## Discussion

In this national sample of 2,633 pediatric patients undergoing posterior spinal fusion, thrombotic events (DVTs, PEs or strokes) were rare, and not associated with TXA use. Additionally, despite concerns that TXA could lead to excitation of neural tissues and prior reports showing seizures resulting from TXA use [30, 31], we did not find evidence of increased seizures in patients who received the drug. Overall, this data supports the safety of TXA in this pediatric population.

The reporting on TXA use in pediatric spine cases is increasing, including studies examining safety [18–21, 38–43]. While our literature search revealed multiple studies on TXA use in pediatric spine surgery, the majority of these, including two large retrospective cohort studies (N > 1,500), limited their findings to TXA efficacy in decreasing blood loss without analyzing adverse events [16, 17]. Out of the trials with a focus on safety of TXA use, all had limited sample sizes which ranged from 44 to 166 patients [18, 20–22, 24]. Meanwhile, research supporting TXA efficacy and safety in adult spine surgery has also been increasing [44, 45]. Multiple recent studies, including one randomized control trial of 68 thoracic spine stenosis patients and a retrospective analysis of 122 patients undergoing lumbar fusion, did not find any associated side effects with TXA use including the incidence of DVTs [46, 47]. Our findings are in line with this growing body of work demonstrating safety of TXA use in spine surgery.

The strength of our study is its large sample size, sampling of data from generalizable everyday practice, and focus on pediatric spine fusion patients. Our large sample size, multiple times larger than prior trials, allowed us to observe even rare side effects which may have been missed in smaller studies and demonstrate that TXA does not increase these risks.

Our findings must be interpreted with limitations. First, this work relied upon administrative data, which can have errors in the coding of diagnoses and procedures. We used previously published or validated code algorithms whenever possible to identify procedures, primary diagnoses, and adverse events in an attempt to mitigate this. However, the code for seizures has not been previously validated for sensitivity and specificity. Second, the new onset diagnosis code modifiers, used to determine if the adverse event occurred perioperatively instead of being a pre-existing condition, have not been specifically validated. As a result, it is possible that adverse events during the admission were undercounted for all patients. Additionally, the baseline and outcome measures which we were able to compare between the control and treatment groups were limited by the database, which did not include data such as the duration of surgery or surgeon experience. These effects may be confounders to our results.

Another surprising finding is that only a minority of patients received TXA in our study. However, this is in line with other large retrospective pediatric studies of TXA efficacy in spinal surgery, where 7% to 30% of the 4,269 and 1,769 patients received TXA respectively [16, 17]. Furthermore, in the context of trauma, this discrepancy was even more pronounced in our data, suggesting hesitation in TXA administration for this patient population. Other studies including a survey of pediatric trauma centers and a study of pediatric patients injured in a combat setting demonstrated similar findings where only a minority of the patients, 35% and 10% respectively were given TXA [48, 49]. This is despite previous research including the CRASH-2 trial demonstrating benefits of TXA use for adult trauma patients [50].

Despite these limitations, we feel that this work provides reassurance to surgeons and perioperative care teams incorporating TXA for the minimization of blood loss during posterior spinal fusion in pediatric patients.

## Conclusions

In this study, TXA use was not associated with an increased risk of thrombotic events (pulmonary embolism, deep vein thrombosis or stroke) and did not precipitate any seizures. Our work demonstrates that the drug is relatively safe to use in the pediatric population for spinal surgery.

## Data Availability

The dataset analysed during the current study is not able to be shared by the authors, due to terms specified by the organization providing the data in a data use agreement. However, the data are available for purchase by the public from Premier Inc. (www.premierinc.com).

## Abbreviations

TXA: Tranexamic acid
PE: Pulmonary embolism
DVT: Deep venous thrombosis
AIS: Adolescent idiopathic scoliosis
CCI: Charlson comorbidity index
